# GnRH agonist pretreatment in hormonal endometrial preparation: a comparison of two protocols for frozen embryo transfer outcomes

**DOI:** 10.3389/fmed.2025.1649012

**Published:** 2025-10-01

**Authors:** Xi Yi, Dongmei Tian, Hengli Li, Guanhua Zhou

**Affiliations:** ^1^School of Medical and Life Sciences/Reproductive and Women-Children Hospital, Chengdu University of Traditional Chinese Medicine, Chengdu, China; ^2^Aba State People’s Hospital, Aba, China

**Keywords:** endometriosis, HRT, FET, gonadotropin-releasing hormone agonist, PCOS

## Abstract

**Objective:**

The aim of this study was to compare the effects of different endometrial treatment regimens on the outcome of frozen embryo transfer (FET).

**Methods:**

A retrospective analysis of 1,454 FET cycles was conducted. Subgroups were stratified by primary infertility diagnosis (tubal factor, polycystic ovary syndrome (PCOS), and endometriosis) and by the number of embryos transferred. Multivariable logistic regression was employed to identify factors influencing live birth.

**Results:**

Our study found that endometrial thickness on the day of embryo transfer was thicker in the gonadotropin-releasing hormone agonist (GnRHa)–hormone replacement therapy (HRT) group compared to the HRT group (*p* = 0.000), the levels of estrogen and progesterone in GnRHa–HRT group were lower than HRT group, and there were no statistically significant differences in other baseline characteristics between the two groups. The live birth rate in the single embryo transfer GnRHa–HRT group was higher than that of HRT(OR = 1.489, 95%CI:1.070–2.073). Within the tubal/male factor cohort, spontaneous abortion rate was slightly more frequent in the GnRHa–HRT group (*p* = 0.899). In the PCOS–endometriosis (EMs) subgroup, the spontaneous abortion rate had a decreasing trend, but the difference was not statistically significant (*p* > 0.05). Binary logistic regression analysis further confirmed the negative influence of age on live birth.

**Conclusion:**

The GnRHa–HRT significantly improves live birth rates compared to the conventional HRT in single-embryo transfer cycles. Downregulation protocols may be a priority for patients with endometriosis and single embryo transfer. For patients with PCOS and tubal factors, the HRT program is more cost-effective and takes less time to achieve pregnancy. These findings provide evidence-based guidance for individualized endometrial preparation regimen selection in FET cycles, though the retrospective design and lack of long-term follow-up limit the generalizability. Future prospective randomized controlled trials with larger sample sizes are warranted to validate these conclusions.

## 1 Introduction

The proportion of frozen–thawed embryo transfers has increased dramatically due to advances in vitrification freezing technology (1). The application of frozen embryo transfer (FET) has become increasingly widespread due to multiple factors, including ovarian stimulation protocols under high progesterone conditions, elevated risks of ovarian hyperstimulation syndrome (OHSS), premature progesterone rise before oocyte retrieval, endometrial abnormalities in fresh cycles, and the use of preimplantation genetic screening. FET is no longer merely a supplementary option to fresh embryo transfer; it has evolved into a standard practice in IVF treatment (2, 3). In FET protocols, three main approaches are employed for endometrial preparation: standard hormone replacement therapy (HRT), gonadotropin-releasing hormone agonist (GnRHa) agonist-supplemented HRT cycles (GnRHa–HRT), and natural cycle monitoring (NC). The natural cycle protocol is generally reserved for ovulatory patients demonstrating regular menstrual cyclicity. However, this approach may result in premature or anovulation, a risk of mis-synchronization between the embryo and the endometrium, and a higher risk of cycle cancellation due to abnormal ovulation. The HRT cycle is more flexible and convenient. Pretreatment with GnRHa before HRT effectively suppresses premature ovulation, thereby avoiding cycle disruption while synchronizing endometrial development with exogenous progesterone administration (4). However, the impact of GnRHa on pregnancy outcome remains controversial. Research has demonstrated no significant differences between HRT and GnRHa concerning the clinical success rates of conception (5). However, other studies have shown that GnRHa–HRT regimens significantly improve pregnancy outcome (6, 7). To resolve the ongoing debate on whether GnRHa pretreatment improves FET outcomes, we conducted a single-center retrospective analysis.

## 2 Materials and methods

### 2.1 Study population

This is a retrospective cohort study with data derived from clinical information on patients who underwent frozen–thawed embryo transfer at the School of Medical and Life Sciences/Reproductive and Women-Children Hospital, Chengdu University of Traditional Chinese Medicine, from September 2016 to September 2020.

### 2.2 Inclusion criteria and exclusion criteria

*Inclusion criteria*: The etiology of infertility was categorized according to the European Society of Human Reproduction and Embryology (ESHRE) guidelines and included the folllowing: male factor: such as oligoasthenospermia and oligoastheno spermia; tubal factors; uterine fibroids: evaluated based on the location and size of the fibroid; endometriosis: diagnosed via laparoscopy, which reveals typical ectopic lesions (e.g., purplish-blue nodules, adhesions) on the pelvic, ovarian, or peritoneal surfaces, confirmed by pathology; Polycystic ovary syndrome (PCOS): diagnosed according to the 2003 Rotterdam criteria, which require the presence of at least two of the following three conditions: oligo-anovulation, hyperandrogenism, or polycystic ovarian morphology; For those unable to undergo laparoscopic examination, a comprehensive diagnosis is made based on pelvic ultrasound, MRI imaging features (such as ovarian chocolate cysts and deep infiltrative lesions), and clinical symptoms (dysmenorrhea, dyspareunia, infertility, etc.); Unexplained infertility: defined as the exclusion of known causes and continuous infertility for ≥ 12 months. Before transplantation, HRT or GnRHa-HRT is used to prepare the endometrium.

*Exclusion criteria*: (1) History of abnormal endometrial hyperplasia and endometrial tuberculosis; (2) surgical history of uterine adhesions; (3) uterine deformity; (4) endothelial thickness of less than 7.0 mm or abnormal endothelial morphology; and (5) hydrosalpinx.

### 2.3 Grouping strategy

**Figure 1:**
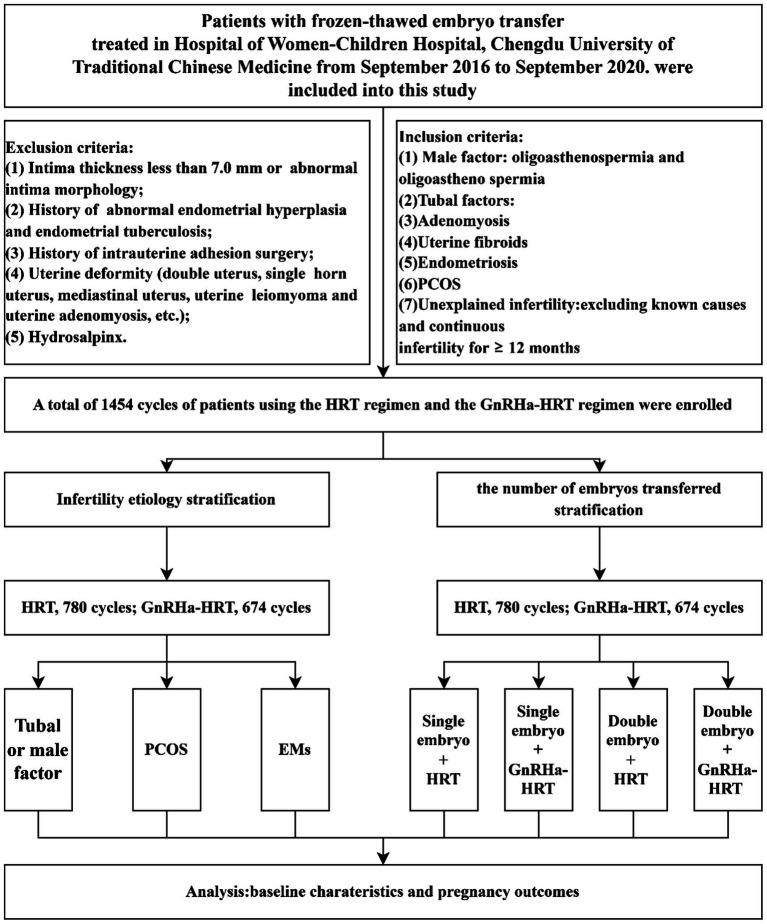
Data collection and analysis. HRT, hormone replacement therapy; GnRHa–HRT, gonadotropin-releasing hormone agonist–HRT; FET, frozen–thawed embryo transplantation; EMs, endometriosis; PCOS, polycystic ovary syndrome.

Study participants were stratified into two cohorts according to GnRHa agonist pretreatment status before HRT: the GnRHa–HRT protocol group and the HRT protocol group. Subsequently, a combined panel of HRT/GnRHa–HRT and the number of transferred embryos stratified patients into four subgroups: Single embryo transfer HRT group, single embryo transfer GnRHa–HRT group, double embryo transfer HRT group, and double embryo transfer GnRHa–HRT group. Based on causes of infertility, the general population was divided into three subgroups: the tubal factor group, the PCOS group, and the endometriosis group. The differences in clinical outcomes between the HRT regimen and the GnRHa–HRT regimen were comparatively analyzed in each of the four cross-stratified subgroups and the three etiologically stratified subgroups described above. This study followed the ethical review requirements of the Declaration of Helsinki. All subjects provided written informed consent before ART-assisted conception, and the anonymity of the patients was protected (Figure 1).

### 2.4 Endometrial preparation protocol

#### 2.4.1 HRT protocol

Starting on menstrual cycle days 2–3, patients take oral Estradiol Valerate tablets (2–4 mg per dose, twice daily) to induce endometrial proliferation and slow follicle development. A transvaginal ultrasound assesses endometrial thickness after 7 days. The Estradiol Valerate dose is adjusted based on the endometrial status, and 17β-estradiol tablets may be added if necessary. Once endometrial thickness reaches ≥7 mm (typically after 14–17 days of Estradiol Valerate), progesterone is started to transform the endometrium and provide luteal phase support. All patients were given a progesterone intramuscular injection 80 mg/day or Progesterone Vaginal Gel 90 g/day transvagina with oral dydrogesterone 20 mg/day. During the cleavage stage embryo transfer, the use of progesterone starts 3 days before transfer. For blastocyst transfer, it starts 5 days before transfer. Pregnancy testing should be performed on day 14 after initiation of progesterone supplementation therapy, and if the test is negative, the combined estrogen and progesterone regimen is terminated. If conception is confirmed, the progestational hormone is continued until 10 weeks of gestation, and the use of estrogen is gradually reduced.

#### 2.4.2 GnRHa–HRT protocol

**Figure 2:**
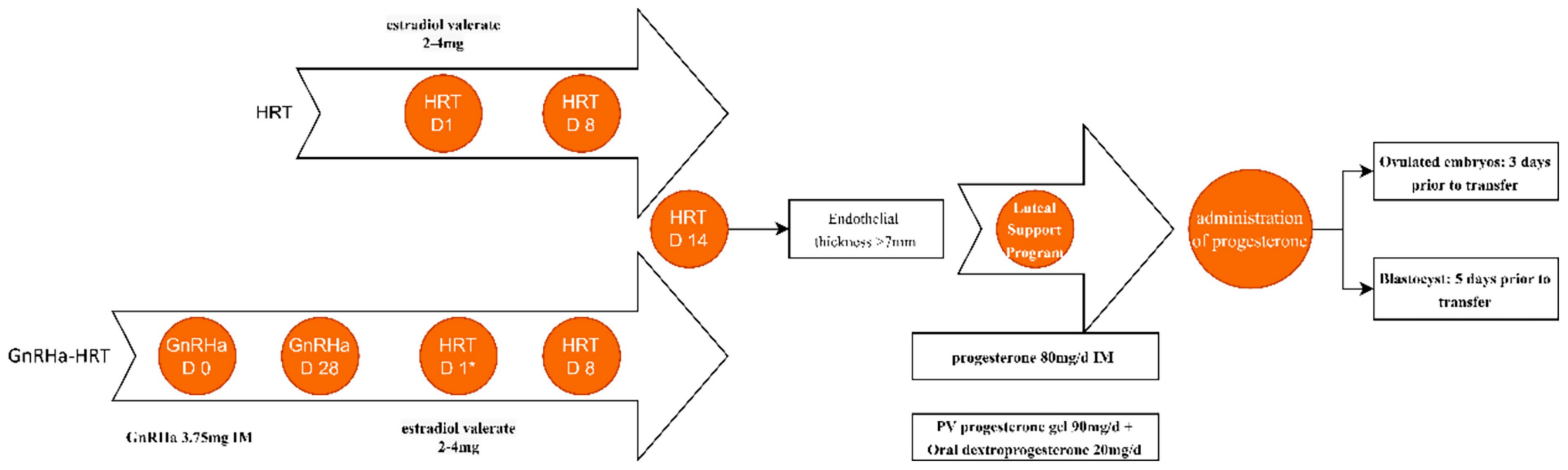
HRT protocols for endometrial preparation. It is usually started on menstrual bleeding day 2–3, which is presented as HRT D1 in the HRT conventional protocol. *HRT D1 is on the 28th day after the second injection. IM, intramuscular. pv, per vagina. Estradiol valerate tablets at a dose of 2–4 mg each time, twice per day.

On menstrual cycle days 2–3, patients receive a 3.75 mg intramuscular GnRHa injection. After 28 days, pituitary downregulation is assessed via transvaginal ultrasound and serum hormone levels. Downregulation criteria include: E2 < 50 pg./mL, FSH < 5 U/L, LH < 5 U/L, endometrial thickness < 5 mm, and no large follicles/cysts. Once met, endometrial preparation begins using the above HRT protocol (Figure 2).

### 2.5 Outcome indicators

The outcomes we analyzed were positive-HCG, clinical pregnancy rate, implantation rate, spontaneous abortion rate, live birth rate, and preterm birth. Secondary objectives include endometrial thickness on the day of ET, serum estrogen level on the ET day, progesterone level on the ET day, and progestin–estrogen ratio (P/E2). Biochemical pregnancy confirmation required quantitative serum *β*-hCG levels exceeding 5.8 IU/L on post-transfer day 14. Ultrasonographic verification of viable intrauterine gestation mandated visualization of gestational sac(s) during the fourth week post-FET sonographic evaluation. Pregnancy loss encompasses non-viable clinical pregnancies occurring before 28 gestational weeks. A live birth is characterized as a pregnancy that progresses with a viable fetus beyond 28 weeks of gestation. Preterm birth was defined as delivery occurring before 37 weeks of gestation. An ectopic pregnancy is characterized by the presence of gestational sacs observed on ultrasound outside the uterine cavity, along with a positive result for serum *β*-hCG. Progestin–estrogen ratio (P/E2) is calculated as P(mg/mL) × 1,000/E2(pg/mL).

### 2.6 Statistical analysis

SPSS 21.0 statistical software was used to analyze the data of this study. Means (standard deviations) were used to describe and compare the numerical variables, and *t*-tests or ANOVAs were used to compare the groups. Numerical values that do not follow a normal distribution are presented as the median along with the interquartile range, and a non-parametric test was conducted to assess the differences between the two groups. Discrete variables were quantitatively characterized through count metrics and relative frequencies (%), with intergroup disparities assessed via χ^2^ contingency analysis. Probability values attaining the critical threshold (*p* < 0.05) served as significance indicators in hypothesis testing. In the four stratified subgroups, the association between different frozen embryo preparation protocols and live birth rates in patients undergoing single and double embryo transfer was analyzed. The ratio of ratios (OR) and their 95% confidence intervals were calculated. Meanwhile, for the endometriosis group, multivariate logistic regression analyses were performed to investigate the factors that influence live birth outcomes. The goodness of fit of the model was evaluated by the Hosmer–Lemeshow test to ensure model stability. Statistical significance was considered when the *p*-value was less than 0.05.

## 3 Results

### 3.1 Overall process analysis

A total of 1,454 cycles with GnRHa pretreatment and HRT cycles were retrospectively analyzed. The initial characteristics of the patients were examined, revealing no substantial differences regarding maternal age, infertility type, infertility duration, and the type of embryo. Estrogen and progesterone levels were found to be higher in the HRT group than in the gonadotropin-releasing hormone analogue–HRT group on the day of transplantation in all patients studied (*p* < 0.05). Additionally, the progesterone-to-estrogen ratio in the HRT group was notably lower than in the GnRHa–HRT group, with all differences showing statistical significance (*p* < 0.05). On the day of the embryo transfer, the endometrial thickness was significantly lower in the HRT group than in the GnRHa–HRT group (*p* = 0.000). Positive-HCG in the HRT group was lower than in the GnRHa–HRT group (53.08% vs. 58.75%, *p* = 0.03), and implantation rate was lower than in the GnRHa–HRT group (35.28% vs. 37.44%, *p* = 0.279). In addition, there was no significant difference in clinical pregnancy rate, spontaneous abortion rate, ectopic pregnancy rate, live birth rate, and preterm birth rate (*p* > 0.05) (Table 1).

**Table 1:** Baseline characteristics and cycle outcomes.

Variables	HRT (*n* = 780)	GnRHa-HRT (*n* = 674)	*p* value
Age (years, mean ± SD)	32.52 ± 4.91	32.84 ± 4.67	0.202
Infertility type (*n*, %)			0.622
Primary infertility (*n*, %)	351 (45.00%)	312 (46.29%)	
Secondary infertility (*n*, %)	429 (55.00%)	362 (55.89%)	
Duration of infertility (years, mean ± SD)	5.35 ± 3.87	5.61 ± 3.89	0.210
Endometrial thickness (mm, mean ± SD)	8.44 ± 1.30	9.09 ± 1.64	0.000
Estradiol level on the day of embryo transfer (pg/ml, Median(Q25, Q75))	169.00 (119.45, 224.40)	138.30 (96.56, 195.25)	0.000
Progesterone level on embryo transfer day (ng/ml, Median(Q25, Q75))	16.14 (11.64, 21.38)	14.97 (10.73, 20.31)	0.001
P/E2 (Median(Q25, Q75))	92.69 (58.74, 148.68)	105.98 (66.39, 168.02)	0.002
No. of transferred embryos (*n*, mean ± SD)	1.60 ± 0.51	1.59 ± 0.49	0.887
Type of embryo transferred (*n*, %)			0.758
Cleavage stage (*n*, %)	403 (51.67%)	361 (53.56%)	
Blastocyst stage (*n*, %)	377 (48.33%)	313 (46.44%)	
β-hCG positive rate (*n*, %)	414 (53.08%)	396 (58.75%)	0.03*
Clinical pregnancy rate (*n*,%)	369 (47.31%)	337 (50.00%)	0.306
Implantation rate (*n*,%)	440/1247 (35.28%)	407/1087 (37.44%)	0.279
Live birth rate (*n*,%)	317 (40.64%)	294 (43.62%)	0.251
Preterm birth rate (*n*,%)	71 (22.40%)	56 (19.04%)	0308
Miscarriage rate (*n*,%)	43 (11.65%)	35 (10.39%)	0.592
Ectopic pregnancy rate (*n*,%)	9 (2.44%)	8 (2.37%)	0.955

HRT, hormone replacement therapy; GnRHa-HRT, gonadotropin-releasing hormone agonist–HRT; P/E2, progesterone/Estradiol, *p < 0.05.

### 3.2 Endothelial preparation protocols cross-stratified with embryo transfer strategies

**Figure 3:**
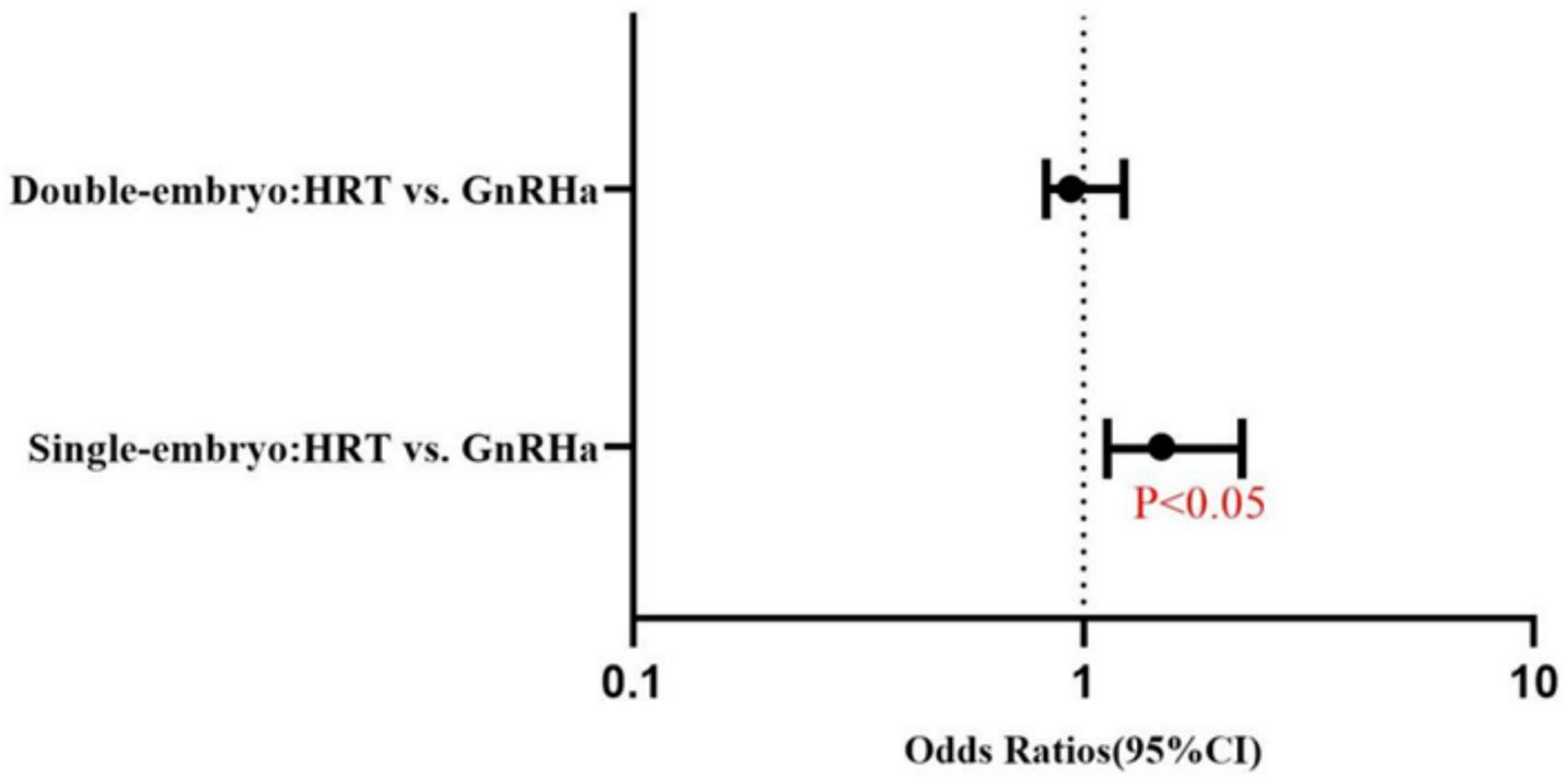
Forest plot of adjusted odds ratios (95% CI) for live birth rates: HRT vs. GnRHa–HRT regimens in single and double embryo transfer (Logistic Regression Analysis). Solid dots represent odds ratios. The error bars were manually drawn based on 95% confidence interval (95% CI) data from the table.

The live birth rate was higher for single embryo transfer with GnRHa–HRT (45.42%) than for single embryo transfer with HRT (35.85%). The difference is statistically significant (OR = 1.489, 95%CI:1.070–2.073). The live birth rates of the GnRHa–HRT group and the HRT group were 43.94 and 42.39% in double embryo transfer. The two groups were indistinguishable from a statistical standpoint (OR = 0.938, 95% CI: 0.715–1.231) (Table 2) (Figure 3).

**Table 2:** Live birth rates and odds ratios of HRT vs. GnRHa–HRT regimens in single and double embryo transfer subgroups.

Subgroup	Regimen	Live birth rate	OR (95%CI)	*p* value
Single embryo transfer (*n*, %)	HRT group	114/318 (35.85%)	1.489 (1.070, 2.073)	0.018
Single embryo transfer (*n*, %)	GnRHa–HRT group	124/273 (45.42%)		
Double embryo transfer (n, %)	HRT group	203/462 (43.94%)	0.938 (0.715, 1.231)	0.645
Double embryo transfer (*n*, %)	GnRHa–HRT group	170/401 (42.39%)		

HRT, hormone replacement therapy; GnRHa-HRT, gonadotropin-releasing hormone agonist-HRT; OR, Odds Ratios; CI, confidence interval.

### 3.3 Stratified analysis according to etiologies

#### 3.3.1 Tubal or male factors group

A total of 951 FET cycles were analyzed: 550 were HRT cycles and 401 were GnRHa–HRT cycles. There were no statistically significant differences observed in maternal age, type of infertility, body mass index, duration of infertility, number of embryos transferred, and type of embryo between the two groups. On the day of embryo transfer, the levels of estrogen and progesterone in the HRT group were significantly higher than in the GnRHa–HRT group (*p* < 0.05). The progesterone-to-estrogen ratio in the HRT group was lower than in the GnRHa–HRT group, with all differences showing statistical significance (*p* = 0.004). On the day of embryo transfer, the endometrial thickness in the HRT group was lower than in the GnRHa–HRT group (*p* = 0.000). In addition, there was no statistically significant difference in positive-HCG, clinical pregnancy rate, spontaneous abortion rate, ectopic pregnancy rate, live birth rate, and preterm birth rate (*p* > 0.05). The rate of spontaneous abortion was higher in the GnRH–HRT group than in the HRT group (10.66% vs. 10.61%, *p* = 0.899), as shown in Table 3.

**Table 3:** Tubal factors or male factors group.

Variables	HRT (*n* = 550)	GnRHa-HRT (*n* = 401)	*p* value
Age (years, mean ± SD)	32.97 ± 5.08	33.62 ± 4.77	0.058
Infertility type (*n*, %)			0.554
Primary infertility (*n*, %)	224 (40.73%)	171 (42.64%)	
Secondary infertility (*n*, %)	326 (59.27%)	230 (57.36%)	
Duration of infertility (years, mean ± SD)	5.49 ± 3.92	5.98 ± 4.19	0.067
Endometrial thickness (mm, mean ± SD)	8.48 ± 1.31	9.26 ± 1.78	0.000
Estradiol level on the day of embryo transfer (pg/ml, Median(Q25, Q75))	167.00 (116.70, 242.70)	134.30 (91.33, 191.40)	0.000
Progesterone level on embryo transfer day (ng/ml, Median(Q25, Q75))	15.92 (0.11.64, 21.75)	14.75 (10.48, 20.36)	0.005
P/E2 (Median(Q25, Q75))	93.81 (59.97, 153.89)	109.90 (68.11, 176.82)	0.004
No. of transferred embryos (n, mean ± SD)	1.61 ± 0.50	1.59 ± 0.49	0.716
Type of embryo transferred (*n*, %)			0.708
Cleavage stage (*n*, %)	295 (53.64%)	220 (54.86%)	
Blastocyst stage (*n*, %)	255 (46.36%)	181 (45.14%)	
β-hCG positive rate (*n*, %)	278 (50.55%%)	217 (54.11%)	0.277
Clinical pregnancy rate (*n*, %)	244 (44.36%%)	179 (44.64%)	0.933
Implantation rate (n, %)	289/883 (32.73%)	209/639 (32.71%)	0.993
Live birth rate (*n*, %)	209 (38.00%)	154 (38.40%)	0.899
Preterm birth rate (*n*, %)	44 (21.05%)	17 (11.04%)	0.012
Miscarriage rate (*n*, %)	26 (10.66%)	19 (10.61%)	0.989
Ectopic pregnancy rate (*n*, %)	8 (3.28%)	6 (3.35%)	0.967

HRT, hormone replacement therapy; GnRHa-HRT, gonadotropin-releasing hormone agonist-HRT; P/E2, Progesterone/ Estradiol.

#### 3.3.2 PCOS factors group

When it came to maternal age, type of infertility, duration of infertility, embryo type, and number of transferred embryos, there were no significant differences. On the day of embryo transfer, the estrogen levels in the HRT group were significantly higher than in the GnRHa–HRT group (*p* < 0.005). There were no statistically significant differences in terms of progesterone levels and the progesterone-to-estrogen ratio (*p* > 0.05). On the day of embryo transfer, the endometrial thickness in the HRT group was lower than in the GnRHa–HRT group (*p* = 0.006). The positive rate of *β*-HCG, clinical pregnancy rate, implantation rate, and live birth rate showed an increasing trend in the GnRHa–HRT group, and the GnRHa–HRT group showed a decrease in spontaneous abortions. There was no statistically significant difference between the two groups (*p* > 0.05), as shown in Table 4.

**Table 4:** PCOS factors group.

Variables	HRT (*n* = 183)	GnRHa-HRT (*n* = 166)	*p* value
Age (years, mean ± SD)	30.86 ± 4.01	30.42 ± 3.68	0.286
Infertility type (*n*, %)			0.643
Primary infertility (*n*, %)	108 (59.02%)	102 (61.45%)	
Secondary infertility (*n*, %)	75 (40.98%)	64 (38.55%)	
Duration of infertility (years, mean ± SD)	4.76 ± 3.32	5.05 ± 2.94	0.391
Endometrial thickness (mm, mean ± SD)	8.22 ± 1.19	8.57 ± 1.16	0.006
Estradiol level on the day of embryo transfer (pg/ml, Median(Q25, Q75))	185.60 (134.80, 267.60)	151.20 (110.70, 206.90)	0.000
Progesterone level on embryo transfer day (ng/ml, Median(Q25, Q75))	16.58 (12.03, 21.19)	15.91 (10.50, 20.75)	0.097
P/E2 (Median(Q25, Q75))	87.49 (51.03, 137.27)	97.92 (62.12, 157.51)	0.087
No. of transferred embryos (n, mean±SD)	1.61 ± 0.53	1.59 ± 0.49	0.694
Type of embryo transferred (*n*, %)			0.619
Cleavage stage (*n*, %)	82 (44.81%)	70 (42.17%)	
Blastocyst stage (*n*, %)	101 (55.19%)	96 (57.83%)	
Biochemical pregnancy (*n*, %)	118 (64.48%)	118 (71.08%)	0.188
Clinical pregnancy rate (*n*, %)	108 (59.02%)	102 (61.45%)	0.643
Implantation rate (*n*, %)	131/295 (44.41%)	133/267 (49.81%)	0.200
Live birth rate (*n*, %)	93 (50.82%)	91 (54.82%)	0.455
Preterm birth rate (*n*, %)	24 (25.81%)	34 (37.36%)	0.092
Miscarriage rate (*n*, %)	14 (12.96%)	10 (9.80%)	0.472
Ectopic pregnancy rate (*n*, %)	1 (0.93%)	1 (0.98%)	0.968

HRT, hormone replacement therapy; GnRHa-HRT, gonadotropin-releasing hormone agonist-HRT; P/E2, Progesterone/ Estradiol.

#### 3.3.3 Endometriosis factors group

##### 3.3.3.1 Compare the pregnancy outcomes between endometrial preparation protocols

No significant differences were observed in terms of maternal age, type of infertility, duration of infertility, type of embryo, and the number of embryos transferred. The estrogen and progesterone levels and the progesterone-to-estrogen ratio on the day of embryo transfer were not significantly different (*p* > 0.05). On the day of embryo transfer, the endometrial thickness in the HRT group was lower than in the GnRHa–HRT group (*p* = 0.084). The positive rate of *β*-HCG, clinical pregnancy rate, implantation rate, and live birth rate showed an increasing trend in the GnRHa–HRT group, and the GnRHa–HRT group showed a decrease in spontaneous abortions. There was no statistically significant difference between the two groups (p > 0.05), as shown in Table 5.

**Table 5:** Endometriosis factors group.

Variables	HRT (*n* = 47)	GnRHa+HRT(*n* = 107)	*p* value
Age (years, mean ± SD)	33.72 ± 4.79	33.80 ± 4.21	0.917
Infertility type (*n*, %)			0.639
Primary infertility (*n*, %)	19 (40.43%)	39 (36.45%)	
Secondary infertility (*n*, %)	28 (59.57%)	68 (63.55%)	
Duration of infertility (year mean ± SD)	6.33 ± 5.06	5.19 ± 3.87	0.131
Endometrial thickness (mm, mean ± SD)	8.78 ± 1.52	9.25 ± 1.58	0.084
Estradiol level on the day of embryo transfer (pg/ml, Median(Q25, Q75))	142.40 (96.38, 204.30)	156.10 (109.58, 326.18)	0.394
Progesterone level on embryo transfer day (ng/ml, Median(Q25, Q75))	15.69 (9.97, 21.13)	13.62 (11.18, 19.17)	0.752
P/E2 (Median(Q25, Q75))	105.61 (70.90, 157.87)	91.54 (58.26, 141.72)	0.298
No. of transferred embryos (*n*, mean ± SD)	1.47 ± 0.50	1.58 ± 0.50	0.204
Type of embryo transferred (*n*, %)			0.192
Cleavage stage (*n*, %)	26 (55.32%)	71 (66.36%)	
Blastocyst stage (*n*, %)	21 (44.68%)	36 (33.64%)	
Biochemical pregnancy (*n*, %)	14 (38.30%)	34 (57.01%)	0.806
Clinical pregnancy rate (*n*, %)	16 (34.04%)	56 (52.34%)	0.036
Implantation rate (*n*, %)	20/69 (28.99%)	65/181 (35.91%)	0.301
Live birth rate (*n*, %)	13 (27.66%)	49 (45.79%)	0.035
Preterm birth rate (*n*, %)	3 (23.08%)	5 (10.20%)	0.218
Miscarriage rate (*n*, %)	3 (18.75%)	6 (10.71%)	0.391
Ectopic pregnancy rate (*n*, %)	0	1 (1.79%)	0.590

HRT, hormone replacement therapy; GnRHa-HRT, gonadotropin-releasing hormone agonist-HRT; P/E2, Progesterone/ Estradiol.

##### 3.3.3.2 Impact of endometrial preparation and clinical factors on live birth logistic regression

To further clarify the impact of the hormone replacement endometrial preparation protocol on the outcome of frozen–thawed embryo transfer, we conducted a correction for confounding factors and conducted the study in the endometriosis group. Binary logistic regression analysis further confirmed that age is an independent risk factor for live birth outcomes in patients with endometriosis (OR = 0.883, 95%CI:0.806–0.966, *p* = 0.007). GnRHa–HRT displayed a trend of increasing the live birth rate (OR = 2.125, 95%CI:0.948–4.762, *p* = 0.067), but the difference did not reach statistical significance. The endometrial thickness (*p* = 0.161) and duration of infertility (*p* = 0.275) had no significant impact on the live birth outcome. The P/E2 ratio shows a potential positive trend (*p* = 0.060), and further validation with a larger sample size is required (Table 6).

**Table 6:** Association between confounding factors and live birth outcomes: results of binary logistic regression analysis.

Confounding factors	OR (95%CI)	*p* value
Endometrial preparation protocol (1)	2.125 (0.948, 4.762)	0.067
Age	0.883 (0.806, 0.966)	0.007
Endometrial thickness	1.176 (0.937, 1.476)	0.161
P/E2	1.004 (1.000, 1.009)	0.060
Duration of infertility	0.944 (0.852, 1.047)	0.275

OR, Odds Ratios; CI, confidence interval; P/E2, Progesterone/ Estradiol.

## 4 Discussion

This retrospective study explored the effect of HRT Protocol and GnRHa–HRT protocol on pregnancy outcomes in FET. Our study found that the proportion of positive *β*-HCG results in the GnRHa–HRT group was significantly higher than in the HRT group across the whole population. This suggests that it has potential advantages in the early stage of embryo implantation and may be related to the optimized endometrial receptivity after downregulation. Downregulation promotes embryo implantation by regulating the expression levels of hormone levels, especially by improving endometrial thickness and the estrogen–progesterone environment (8, 9). However, there were no significant differences in the rates of clinical pregnancy and live birth. It has been demonstrated that, while the downregulation can promote early implantation, the maintenance of subsequent pregnancies is governed by a combination of various factors (such as embryo quality and the maternal immune status). Therefore, clinical judgment should not solely rely on the positive rate of *β*-HCG.

The endometrial thickness on the day of embryo transfer in the HRT group was less than that in the GnRHa–HRT group. This was statistically significant (*p* = 0.006). The progesterone/estradiol ratio was higher than that in the HRT group. The main reason is that GnRHa downregulation leads to the inhibition of the HPO axis, reducing the basal estrogen level in the body and the basal estrogen level in the uterine tissue. Some research has shown that estrogen is a permissive rather than a dose-dependent regulator of endometrial development (10), and so the amount of estrogen required during the implantation period is extremely low. Studies find that high estrogen levels during pregnancy harm the endometrium. First, high levels of estrogen have a negative feedback effect on LH, leading to impaired luteal function and affecting endometrial tolerance (11). Elevated serum E2 concentrations may impair the endometrial lining, reduce the time frame for potential implantation, and hinder the embedding of the embryo in the endometrium (12). Finally, high estrogen may inhibit the emergence of cytosolic synapses and block the response of cytosolic synapses to progesterone (13, 14). This may be the reason why the GnRHa–HRT regimen can improve endometrial tolerance by lowering estrogen levels. A study by E. Shalom-Paz et al. on fresh cycle patients found that P/E2 on the day of HCG injection was significantly lower in the live birth group than in the no live birth group (15). On the day of embryo transfer, the endometrial thickness was found to be thinner in the HRT group than in the GnRHa–HRT group (*p* = 0.006). Further studies are needed to confirm the effect of P/E2 on pregnancy outcome in frozen embryo cycles.

Hao et al. (8) found that the proportion of single embryo transfers was significantly higher in the GnRHa–HRT group. However, the study did not compare GnRHa–HRT pregnancy outcomes with HRT pregnancy outcomes by dividing single/ double embryo transfer. Our study found that the GnRHa–HRT significantly improves live birth rates compared to the conventional HRT in single-embryo transfer cycles. A statistically significant difference was found between the two groups (OR = 1.489; 95% CI: 1.070–2.073). Our finding of a higher rate of singleton pregnancy in the GnRHa–HRT group suggests a potential advantage of this protocol in achieving singleton pregnancies, which could help to meet the clinical goal of reducing multiple pregnancy risk.

Our study found that the GnRHa–HRT regimen did not yield statistically significant benefits in patients with polycystic ovary syndrome (PCOS) or endometriosis (EMs) (*p* > 0.05), but we observed a trend toward improvement in certain clinical outcome measures. This may be related to a specific mechanism in patients with PCOS and EMs. In this study, we found that GnRHa–HRT after GnRHa downregulation significantly increased the live birth rate in patients with endometriosis (42.1% vs. 21.6%, *p* = 0.035). Previous studies also suggest that GnRHa pretreatment was shown to improve the reproductive outcomes of patients with endometriosis (16). There may be the following mechanisms: (1) There are GnRHaa receptors on the endometrium. After GnRHa binds to these receptors, it can downregulate the expression of TGF-*β* and its receptors, inhibit the phosphorylation of Smad3 and its translocation from the cytosol to the nucleus, thereby reducing the deposition of extracellular matrix and inhibiting the formation of fibrous adhesions (17); (2) GnRHa-a can promote the expression of key transcription factors such as LIF, MEIS1, and HOXA10 (18, 19), enhancing the expression of endometrial αvβ3 integrin, increasing the number of pinopodes, and increasing number of cilia, thereby ultimately improving endometrial receptivity (18, 20–24). Furthermore, GnRHa can also enhance the expression of IL-6 and IL-11 mRNA regulated by miR-124-3p in endometrial stromal cells, enhancing endometrial receptivity (25, 26); (3) GnRHa can inhibit local inflammatory response and downregulate cytotoxicity of NK cells to improve the intrauterine environment, thereby improving embryo implantation (27, 28). Furthermore, GnRHa–HRT can reduce the expression of the proinflammatory factors IL-6 and IL-11, which may help increase the rate of childbirth (25). GnRHa may also break down the balance between Treg and Th17 cells and exert a direct immunomodulatory effect, thereby enhancing the receptivity of the endometrium (29) (4). Other studies have demonstrated that GnRHa–HRT can reduce nitric oxide synthase expression of endometrium (30). However, a study found that downregulation is only effective for patients with stage III or IV endometriosis, but ineffective for those with mild endometriosis (26). Therefore, in the future, high-quality data will still be needed to support this argument.

Current research has found that clinical pregnancy outcomes in patients with PCOS treated with GnRHa–HRT are controversial. Some studies found that using long-acting GnRHa for pretreatment resulted in an improvement in clinical pregnancy rates in patients with PCOS (31, 32). However, some studies have shown that GnRHa–HRT pretreatment does not improve pregnancy outcomes in patients with PCOS (31, 32). Although we found an improvement in the endometrial thickness of PCOS patients, it did not lead to an improvement in the pregnancy outcome. The possible mechanism is that the complex pathology of PCOS patients (e.g., metabolic disorders, oocyte quality defects) may counteract the endothelial optimization effect of downregulation (33, 34). Therefore, one needs to weigh the potential benefits against the risks when using GnRHa. Although the endometrial thickness of the GnRHa–HRT group has increased in the Tubal factors group, the levels of estrogen and progesterone decreased, and the incidence of premature birth significantly increased. The possible mechanism is that the hormonal imbalance caused by the lowering of the pitch leads to cervical insufficiency or abnormal placental function. It suggests that when using a lowering treatment plan for such patients, the risks need to be carefully evaluated.

The results of the logistic regression analysis indicated that age was a standalone risk factor for live birth, consistent with previous studies (35, 36). Endothelial preparation regimens were borderline positively associated with live birth outcomes (*p* = 0.067), which, although not at the traditional level of significance, combined with the results of the subgroup analyses, strongly suggests the potential value of downregulation regimens in specific populations. This “borderline significant” result may also be limited by the single-center nature of the study, insufficient sample size, or confounding factors that were not fully controlled for (e.g., individual patient differences, details of the ovulation regimen).

This study has certain limitations. First, as a retrospective single-center study, it may be subject to selection bias. Second, etiologic stratification was not further refined (e.g., metabolic subtypes of PCOS and lesion types of endometriosis), which might have affected the assessment. Third, some potential confounding variables (e.g., lifestyle, embryo quality) were not included in the analysis; notably, the lack of control for embryo quality—a key factor in transplantation outcomes—may impact result interpretation. Additionally, although patients were fully informed about differences in cost (e.g., medication, number of visits) and time-to-pregnancy (e.g., extended duration due to GnRHa downregulation in GnRHa–HRT), during clinical decision-making, this study did not systematically collect or analyze such data, limiting comprehensive evaluation of economic and time efficiency. In subsequent research, we will refine embryo quality assessment and integrate it into analytical models. Future multicenter prospective studies should combine molecular marker testing, precise etiologic typing, cost-effectiveness analysis, and time-to-pregnancy tracking to explore mechanisms of different endometrial preparation regimens, providing stronger evidence for individualized treatment.

## 5 Conclusion

In summary, this study confirms the critical impact of age on pregnancy outcomes and reveals the differential effects of a downregulation program across various populations. In clinical practice, endothelial preparation regimens should be selected comprehensively according to patient etiology, transplantation strategy, and other relevant factors. For patients with endometriosis undergoing single-embryo transfer, the downregulation regimen should be considered a priority. In contrast, for patients with PCOS or tubal factor infertility, the advantages and disadvantages of the regimen need to be carefully weighed and applied cautiously to maximize the efficacy of the treatment of assisted reproduction.

## Data Availability

The raw data supporting the conclusions of this article will be made available by the authors, without undue reservation.
